# Kinetic and Spectroscopic Evaluation of β-Carotene and α-Tocopherol Degradation in Fatty Acid Methyl Esters

**DOI:** 10.3390/molecules31132298

**Published:** 2026-07-01

**Authors:** Paweł Grabowski, Angelika Szwarczyńska, Szymon Skorupski

**Affiliations:** Faculty of Civil Engineering, Mechanics and Petrochemistry, Warsaw University of Technology, Lukasiewicz Street 17, 09-400 Plock, Poland

**Keywords:** fatty acid methyl esters, natural antioxidants, β-carotene, α-tocopherol, oxidative stability

## Abstract

The limited oxidative stability of fatty acid methyl esters (FAME) constrains their use as biofuels. This study presents a direct, side-by-side comparison of β-carotene and α-tocopherol as natural antioxidant additives in FAME produced from refined rapeseed oil, evaluated under identical thermo-oxidative conditions (100–140 °C). We combine kinetic modelling (first-order and zero-order fits, Arrhenius analysis) with spectroscopic monitoring (UV–Vis for β-carotene; FT-IR for α-tocopherol) and standard oxidation indices (PV, AnV) to link antioxidant depletion to fuel oxidation. Key findings are: (1) β-carotene effectively delays hydroperoxide formation at lower temperatures but degrades rapidly above 120 °C (Ea 6–23 kJ·mol^−1^), producing secondary products that increase AnV; (2) α-tocopherol shows greater thermal resistance and predictable, dose-dependent protection across the tested range (optimal at 556 µg·mL^−1^), with higher doses exhibiting potential pro-oxidant effects; (3) activation energies and kinetic orders differ between antioxidants, indicating distinct degradation pathways in the FAME matrix. These results demonstrate that reintroducing natural antioxidants removed during refining can improve biodiesel durability, and that antioxidant selection and dosing must be tailored to expected thermal exposure. The combined kinetic–spectroscopic approach provides a practical framework for optimizing natural additive strategies in biodiesel formulations.

## 1. Introduction

The rapid development of the biofuel sector over the past two decades has been driven by the need to reduce greenhouse gas emissions and diversify energy sources in transport. In the European Union, regulatory frameworks such as the Renewable Energy Directive (RED II, 2018/2001/EU) and its revision RED III (2023/2413/EU) play a central role by setting minimum shares of renewable energy in transport and promoting the use of low-carbon biofuels [1,2]. In this context, fatty acid methyl esters (FAME) remain one of the most important first-generation biofuels, meeting the requirements of the EN 14214 standard [3] and constituting a major component of diesel blends [4].

FAME are produced by transesterification of vegetable oils or animal fats with methanol in the presence of a basic catalyst. The fatty acid composition of the feedstock determines the physicochemical properties of the product, including its susceptibility to oxidation. Rapeseed oil, one of the most widely used feedstocks in Europe, is rich in oleic, linoleic, and linolenic acids, which favor the formation of reactive lipid hydroperoxides during storage and fuel use [5,6,7]. The oxidation mechanism of FAME involves radical initiation, propagation, and termination steps, and its course depends strongly on temperature, oxygen availability, light exposure, and trace transition metals [8,9].

Oxidative stability is a key parameter determining the operational suitability of FAME, as emphasized by Knothe [10]. Oxidation leads to the formation of hydroperoxides, aldehydes, ketones, carboxylic acids, and oligomers, which can cause corrosion of fuel system components, increase viscosity, and promote deposit formation in injectors [11,12]. Elevated temperature accelerates FAME degradation, and peroxide value (PV), anisidine value (AnV), and acid value (AV) are reliable indicators of oxidation progress, complementary to standardized accelerated aging tests such as Rancimat and PetroOXY [13,14,15].

Natural antioxidants, including tocopherols and carotenoids, play an important role in stabilizing lipids in biological and food systems. Tocopherols, particularly α-tocopherol, are among the most effective fat-soluble antioxidants; they act as peroxyl radical scavengers and interrupt chain-propagation steps of lipid oxidation [16,17,18]. β-Carotene, a representative carotenoid, quenches singlet oxygen and neutralizes free radicals due to its conjugated double-bond system [19,20]. Both compounds occur naturally in vegetable oils, but refining processes remove them almost completely, significantly reducing the oxidative resistance of FAME produced from refined oils [21].

Interest in natural antioxidants as stabilizing additives for biofuels has increased in recent years. Studies have shown that tocopherols can substantially extend the induction period of FAME oxidation, limiting both hydroperoxide formation and secondary oxidation products [22,23,24]. β-Carotene exhibits high antioxidant efficacy at lower temperatures; however, above approximately 120 °C it undergoes rapid decomposition, yielding reactive secondary products such as epoxides and carotenoid-derived aldehydes [25,26]. Mechanistic studies indicate the relevance of pathways such as SPLET (sequential proton loss electron transfer) and highlight the strong influence of the reaction environment on carotenoid stability [27].

For biofuel applications, it is particularly important to compare the effectiveness of different natural antioxidants under identical thermo-oxidative conditions. Wang et al. reported that α-tocopherol and β-carotene may exhibit synergistic or antagonistic interactions depending on their ratio and temperature [28]. According to the results reported by [28], tocopherols not only delay the onset of oxidation but also maintain radical-scavenging capacity during prolonged heating, whereas carotenoids undergo rapid structural degradation, limiting their protective effect. Studies on vegetable oil stability indicate that carotenoids act effectively only within a limited range of conditions, whereas tocopherols retain activity over a broader temperature range [29,30]. Previous studies on vegetable oil stability indicate that carotenoids exhibit antioxidant activity primarily at moderate temperatures (typically below 120 °C), whereas their effectiveness decreases rapidly under more severe thermal stress [26]. In contrast, tocopherols retain activity over a broader temperature range, remaining effective up to approximately 150–160 °C, depending on the matrix and oxidation conditions. These findings highlight the distinct thermal behavior of natural antioxidants and justify the need for comparative evaluation under controlled thermo-oxidative conditions [16].

These findings highlight the need for a detailed analysis of the impact of natural antioxidants on the oxidative stability of FAME, especially under high-temperature conditions that may occur during storage, transport, and engine operation. Reintroducing tocopherols and carotenoids into FAME produced from refined rapeseed oil may improve its durability; however, this requires precise determination of their efficacy under different thermal conditions. In the present study, a direct and systematic comparison of β-carotene and α-tocopherol as natural antioxidants stabilizing FAME was performed under identical thermo-oxidative conditions spanning 100–140 °C, a range characteristic of realistic fuel aging processes. This represents a clear scientific novelty, as previous studies examined these compounds separately, in different matrices, or at lower temperatures. Here, simultaneous monitoring of FAME oxidation indices (PV, AnV, AV) and antioxidant degradation kinetics using UV–Vis and FT-IR spectroscopy allowed us to capture their actual protective performance. Additionally, the novelty of this work lies in the direct comparison of β-carotene and α-tocopherol under identical thermo-oxidative conditions in the same FAME matrix, enabling a consistent kinetic and mechanistic evaluation not available in previous studies. Additionally, the study reconstructs the oxidative properties of FAME obtained from refined rapeseed oil—commonly used in households and catering and frequently appearing as waste frying oil in FAME production—demonstrating that restoring natural antioxidants can reduce the need for synthetic additives. In this study, FAME was used as a model oxidation substrate to investigate the thermo-oxidative behavior of β-carotene and α-tocopherol. The aim was not to evaluate fuel quality in the context of EN 14214 compliance, but to analyze antioxidant performance under controlled laboratory conditions.

## 2. Results and Discussion

The study was carried out in two measurement series. In the first series, the effect of β-carotene added to FAME was analyzed, while in the second series α-tocopherol was examined in the same matrix. Synergistic interactions between the antioxidants were not investigated; instead, the focus was placed on the individual mechanisms of action and the thermal sensitivity of each compound. The study was carried out in two measurement series. In the first series, the effect of β-carotene added to FAME was analyzed, while in the second series α-tocopherol was examined in the same matrix. Synergistic interactions between the antioxidants were not investigated; instead, the focus was placed on the individual mechanisms of action and the thermal sensitivity of each compound. Although different kinetic orders were applied, the obtained parameters remain comparable because each model reflects the dominant degradation pathway of the respective antioxidant under identical experimental conditions.

**Figure 1:**
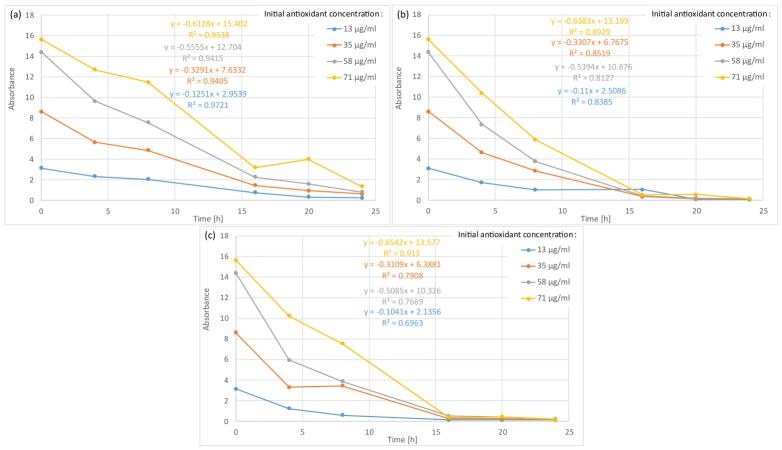
Changes in the absorbance of FAME containing β-carotene with fitted zero-order kinetic equations A = f(t) at (**a**) 100 °C, (**b**) 120 °C, and (**c**) 140 °C (Absorbance is expressed as a dimensionless quantity).

At 100 °C, higher initial β-carotene concentrations (35–71 µg/mL) display a slower relative decrease in absorbance compared with the lowest concentration (13 µg/mL), as shown in Figure 1. This apparent paradox—where absolute slopes (ΔA/Δt) may be larger for higher *A*_0_ while fractional loss *A*/*A*_0_ is slower—can be explained by a transient self-protection (shielding) effect. An initially more labile subpopulation of molecules (for example, surface-accessible chromophores or molecules in oxygen-exposed microenvironments) is consumed first, temporarily reducing the effective oxidation rate experienced by the remaining bulk chromophores. Normalized *A*/*A*_0_ behavior and initial-rate estimates support this interpretation. At elevated temperatures (≥120 °C) the shielding effect is overwhelmed by rapid thermal cleavage and parallel degradation pathways, causing the A(t) curves for different initial concentrations to converge.

At 120 °C, the behavior of the A(t) curves changes markedly. Initially, higher β-carotene concentrations degrade slightly more slowly; however, after 16–24 h all samples reach very low concentration levels, and the differences between them nearly disappear. This indicates that the degradation rate becomes sufficiently high and that the initial concentration no longer influences the reaction course. The A(t) curves become steeper, reflecting intensified degradation processes such as cleavage of the conjugated double-bond system and formation of epoxides and apocarotenals.

At 140 °C, β-carotene degradation is the fastest and occurs almost immediately. After only 4–8 h, differences between initial absorbance values become minimal, and after 24 h all samples reach values close to zero. After 16 h, the A(t) curves for all concentrations nearly overlap, indicating that the degradation process is controlled exclusively by temperature rather than by the amount of β-carotene present. Under these conditions, β-carotene loses its antioxidant activity regardless of dose, and its molecular structure undergoes rapid breakdown. The insufficient fit to zero-order kinetics prompted further analysis to determine the appropriate kinetic model describing β-carotene degradation in FAME.

The absorbance-dependent behavior observed in the β-carotene degradation curves arises from the combined influence of matrix-related effects and the intrinsic multi-pathway nature of carotenoid thermo-oxidation. At lower temperatures, several concurrent factors may contribute to the temporary convergence or irregularity of the A(t) curves, including (i) a transient self-protection or shielding effect caused by preferential consumption of a more labile subpopulation of β-carotene molecules, (ii) inner-filter and optical effects at high initial absorbance, (iii) oxygen-diffusion limitations within the sealed vial, and (iv) microenvironmental heterogeneity or aggregation of carotenoids in the FAME matrix. Superimposed on these matrix effects is the intrinsic chemistry of β-carotene, which undergoes simultaneous isomerization, polyene chain scission and formation of conjugated oxidation intermediates. The relative contribution of these pathways depends on both temperature and concentration, leading to transient increases or non-monotonic changes in absorbance at selected doses (e.g., 35 and 71 µg/mL). At higher temperatures, rapid thermal cleavage dominates, eliminating the transient shielding phenomena and producing more uniform degradation kinetics. This interpretation is consistent with the kinetic fits and the Arrhenius behavior discussed above. Minor initial fluctuations in absorbance at the lowest concentrations are attributed to baseline stabilization and do not affect the overall degradation trend.

**Figure 2:**
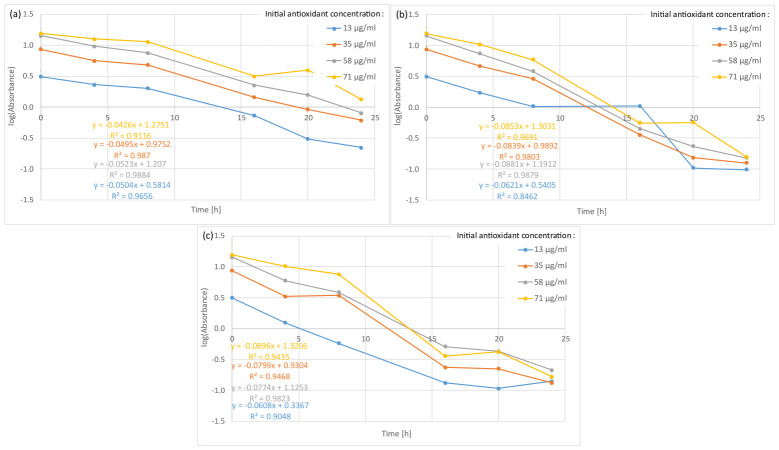
Changes in the absorbance of FAME containing β-carotene with fitted first-order kinetic equations log(A) = f(t) at (**a**) 100 °C, (**b**) 120 °C, and (**c**) 140 °C.

Plots of log(A) as a function of time reveal clear linear relationships supporting the applicability of a first-order kinetic model (Figure 2). Fits with R^2^ > 0.84 and significance levels below 0.0033 confirm the statistical relevance of the results (*p* < 0.05). At 100 °C, the log(A)(t) curves for all concentrations are approximately linear, confirming first-order kinetics. Differences between concentrations remain visible, reflecting the self-protection effect also observed in the A(t) plots.

At 120 °C, the log(A)(t) plots remain linear; however, differences between initial concentrations disappear much more rapidly than at 100 °C. This indicates that the degradation rate becomes independent of the initial antioxidant concentration, while the process continues to follow first-order kinetics until β-carotene is nearly depleted. At 140 °C, the log(A)(t) plots remain approximately linear, although slight deviations appear during the initial hours, particularly for the highest initial concentrations. These deviations arise from the very rapid loss of the conjugated double-bond system and the overlap of several parallel degradation pathways. Nevertheless, first-order behavior remains dominant, and the slopes of the regression lines are the steepest among all temperatures, reflecting the fastest degradation.

A first-order kinetic model was applied using changes in absorbance at 450 nm, corresponding to the β-carotene chromophore. The kinetic equation log(A/A_0_) = −kt was used to determine apparent rate constants k for each temperature. The log(A)(t) plots exhibited very good linearity, confirming the validity of the model. Table 1 presents the calculated rate constants and corresponding half-life values (t_1_/_2_). As temperature increases, k values rise and t_1_/_2_ decreases, demonstrating the accelerating effect of temperature on β-carotene degradation.

**Figure 3:**
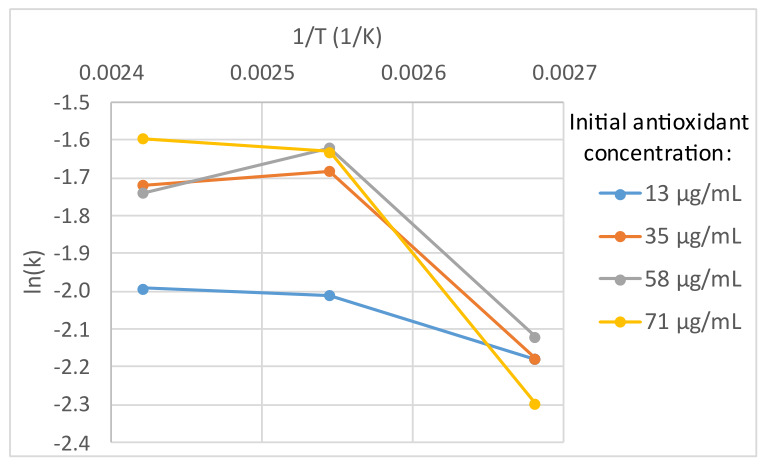
Dependence of the logarithm of the rate constant on the inverse of temperature for the degradation of β-carotene.

The Arrhenius plot (Figure 3) shows a linear dependence of ln(k) on 1/T, enabling determination of the effective activation energy for β-carotene degradation. The linear fits shown in Figure 3 exhibited high coefficients of determination (R^2^) (from 0.56 to 0.84), confirming the adequacy of the applied model despite minor experimental scatter. The obtained activation energies (6–23 kJ/mol) are significantly lower than typical activation energies for biodiesel oxidation, which was reported by Knothe [10] (65–100 kJ/mol) and Encinar et al. [31] (60–120 kJ/mol). This confirms that β-carotene degrades much more readily than the FAME matrix itself and that its protective effect diminishes rapidly under elevated thermal conditions.

These results indicate that β-carotene undergoes facile thermal degradation, and its consumption may occur already at moderately elevated temperatures, before intensive oxidation of methyl esters begins. Therefore, the obtained E_a_ values, shown in Table 2, should be interpreted as parameters describing the stability of the antioxidant within the FAME matrix rather than as activation energies of biodiesel aging. Nevertheless, the degradation rate of β-carotene remains an important factor influencing long-term biodiesel stability, as it determines the duration of effective protection against autoxidation.

**Figure 4:**
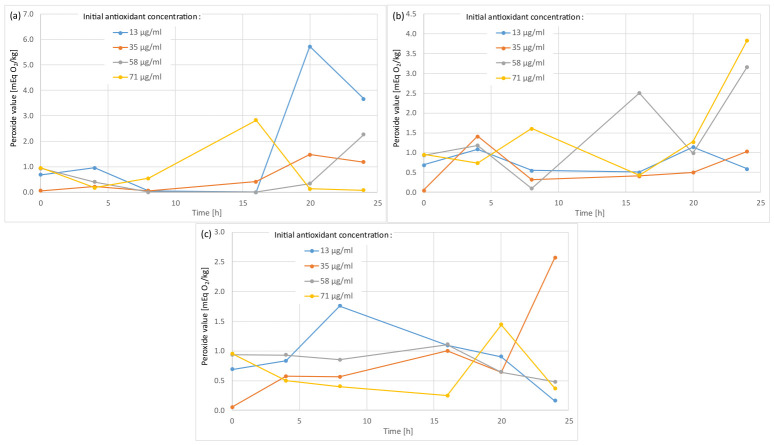
Changes in the peroxide value of FAME containing β-carotene over time at (**a**) 100 °C, (**b**) 120 °C, and (**c**) 140 °C.

At 100 °C, β-carotene slightly suppresses the increase in peroxide value (PV), with PV depending on natural antioxidant concentration (Figure 4). At 120 °C, PV rises more rapidly, and differences between concentrations diminish, indicating limited protection. At 140 °C, PVs are high and similar across all concentrations, confirming that β-carotene loses antioxidant activity almost immediately at high temperature.

Peroxide value (PV) reflects the instantaneous concentration of lipid hydroperoxides, whose net level is governed by competing formation and decomposition processes. Under thermo-oxidative conditions, the balance between hydroperoxide generation, thermal cleavage and antioxidant-mediated pathways can shift during the experiment, producing transient non-monotonic PV traces (local plateaus or decreases followed by increases). We therefore interpret PV changes as a dynamic equilibrium outcome rather than a simple monotonic accumulation. At longer oxidation times, β-carotene is largely consumed, and the rate of hydroperoxide formation becomes controlled primarily by the FAME matrix rather than by the initial antioxidant concentration. Consequently, the PV curves for different β-carotene doses converge, which we describe as “diminishing differences between concentrations”.

**Figure 5:**
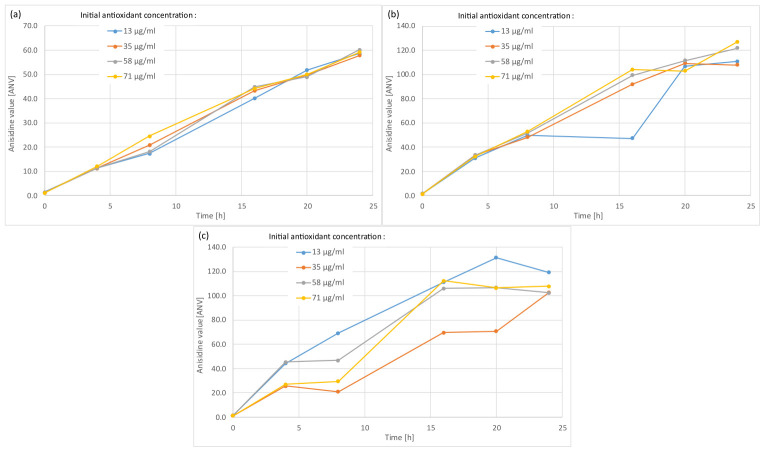
Changes in the anisidine value of FAME containing β-carotene over time at (**a**) 100 °C, (**b**) 120 °C, and (**c**) 140 °C.

Changes in anisidine value (AnV) reveal that at 100 °C, β-carotene partially suppresses the formation of secondary oxidation products (Figure 5). At 120 °C, AnV increases rapidly, and degradation products of β-carotene may exert pro-oxidant effects. At 140 °C, AnV rises quickly and reaches similar values for all concentrations, confirming the lack of protective activity.

At 100 °C, the oxidation rate remains low and the system is still in the induction phase. Under these mild conditions, β-carotene retains its antioxidant activity for an extended period, and its depletion is minimal. As a result, the A(t) curves for all concentrations are very similar, because the antioxidant dose does not yet limit the oxidation rate. The concentration-dependent effects become visible only at higher temperatures, where β-carotene degradation accelerates and the system transitions into the propagation phase of oxidation.

Considering that biodiesel is typically stored at low temperatures, β-carotene may still contribute to preventing oxidation under realistic storage conditions. This behavior can be further explained by the chemical nature and instability of lipid hydroperoxides. The convergence of PV curves arises from the intrinsic instability of hydroperoxides, which undergo rapid thermal cleavage into aldehydes, ketones and acids. Their concentration therefore represents a dynamic equilibrium rather than a linear oxidation trajectory. Once β-carotene is depleted, hydroperoxide formation becomes independent of the initial antioxidant dose, leading to similar PVs across concentrations. This behavior is typical for lipid systems undergoing advanced thermo-oxidation. The similarity of the curves at 100 °C for AV reflects the characteristic behavior of lipid systems in the induction phase. At this stage, hydroperoxide formation is slow, and β-carotene remains largely intact, providing effective radical-quenching activity regardless of its initial concentration. Consequently, the oxidation rate is governed primarily by the intrinsic stability of the FAME matrix rather than by the antioxidant dose. Only at elevated temperatures (≥120 °C), where β-carotene undergoes rapid thermal degradation, do clear concentration-dependent differences emerge. Such non-monotonic behavior (e.g., transient increases followed by decreases) reflects the fact that hydroperoxide concentration is governed by the competing rates of formation and thermal decomposition, which may alternate in dominance during prolonged heating.

According to the literature, the presence of natural pigments such as carotenoids enhances the oxidative stability of oils by increasing the resistance of the lipid matrix to oxidation, whereas their removal shortens the induction period and accelerates degradation [21,32]. Studies on vegetable oils have shown that β-carotene effectively delays the initiation of radical reactions, reduces the rate of hydroperoxide formation, and stabilizes intermediate oxidation products [33]. Since biodiesel is a lipid-based system with analogous susceptibility to autoxidation, the antioxidant behavior of β-carotene can be transferred to FAME. Research on biodiesel stability emphasizes that natural antioxidants present in plant feedstocks—particularly α-tocopherols and carotenoids—are responsible for its initial resistance to oxidation [10,34]. It has also been demonstrated that natural extracts rich in carotenoids can extend the oxidative stability of biodiesel by acting as radical-reaction inhibitors during the early stages of oxidation [13]. Compared with α-tocopherol, however, β-carotene is significantly less stable and less effective, although it plays a role in regenerating α-tocopherol from the tocopheryl radical [20].

In view of these findings, α-tocopherol was examined under the same conditions as β-carotene. Like β-carotene, α-tocopherol exhibits antioxidant activity in lipid systems; however, its degradation pattern differs fundamentally. In this study, changes in the absorbance of the 1068 cm^−1^ FT-IR band, characteristic of C–O vibrations in the α-tocopherol structure, were analyzed. The results revealed a degradation profile markedly different from that observed for β-carotene. This means that the degradation rate remains constant and does not depend on the current concentration of the compound, but is controlled primarily by external conditions—temperature and the properties of the lipid matrix.

**Figure 6:**
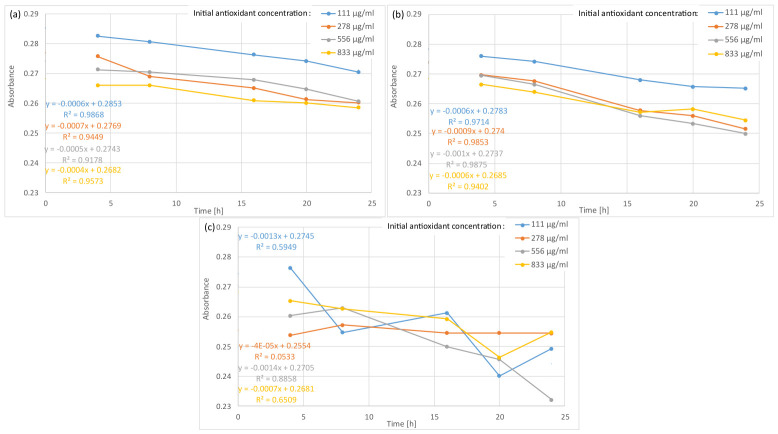
Changes in the absorbance of FAME containing α-tocopherol with fitted zero-order kinetic equations A = f(t) at (**a**) 100 °C, (**b**) 120 °C, and (**c**) 140 °C.

At 100 °C, the absorbance of α-tocopherol decreased slowly and uniformly for all tested concentrations, as was shown in Figure 6. Increasing the temperature to 120 °C accelerated the signal loss, yet the linear character of the A(t) curves was preserved, confirming zero-order behavior. The highest degradation rate was observed at 140 °C, although for the 278 μg/mL sample a partial stabilization of the signal occurred, likely due to matrix effects or overlapping IR bands.

Compared with β-carotene, α-tocopherol exhibits a fundamentally different degradation mechanism. While β-carotene degraded according to first-order kinetics, α-tocopherol followed zero-order kinetics, where the rate-limiting step is the reaction with lipid radicals rather than spontaneous thermal decomposition. Linear model fitting yielded high coefficients of determination (R^2^ > 0.9 at 100 °C and 120 °C), confirming the validity of the zero-order model.

Table 3 presents the rate constants (k) and corresponding half-life values (t_1_/_2_) for α-tocopherol degradation. Calculations were performed separately for each concentration (111, 278, 556, and 833 μg/mL) and temperature (100, 120, and 140 °C), since zero-order kinetics require independent determination of k for each initial absorbance level. The corrected kinetic parameters presented in Table 3 show that the temperature-dependent changes in the rate constant (k) and half-life (t_1_/_2_) do not follow a strictly monotonic trend. Although higher temperatures generally accelerate β-carotene degradation, the values indicate that the reaction kinetics are influenced by several competing pathways and do not scale linearly with temperature or concentration. Higher concentrations exhibited longer half-life values, consistent with the mechanism of phenolic antioxidants, where larger amounts of α-tocopherol extend the time required to reach a 50% decrease in absorbance.

The 278 µg/mL concentration at 140 °C exhibited non-monotonic A(t) behaviour and was therefore excluded from kinetic fitting (see Table 3 note). α-tocopherol does not degrade through a single reaction but through several competing reactions, including hydrogen-atom donation, formation of tocopheroxyl radicals, recombination, and secondary oxidation. The relative contribution of these pathways shifts with temperature and with the extent of lipid oxidation. Moreover, hydroperoxides formed in the matrix undergo statistically driven decomposition, generating reactive species that can either accelerate or transiently inhibit tocopherol loss.

**Figure 7:**
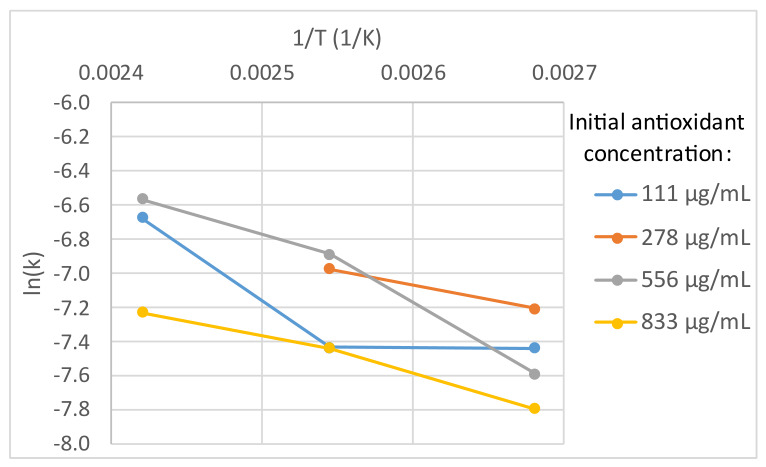
Arrhenius plots (ln k vs. 1/T) for α-tocopherol degradation in FAME. Linear regressions were used to extract activation energies; minor deviations from ideal linearity are discussed in the Kinetics section. The 278 µg/mL point at 140 °C was excluded from fitting due to non-monotonic A(t). R^2^ range: 0.56–0.84.

The Arrhenius plot (Figure 7) illustrates the relationship between ln(k) and 1/T for α-tocopherol degradation. All series exhibit linear behavior, confirming consistency with the Arrhenius equation and indicating that α-tocopherol degradation is a temperature-controlled process. The slopes of the regression lines correspond to activation energies, while vertical offsets reflect differences in the pre-exponential factor. Higher α-tocopherol concentrations show smaller decreases in ln(k) with increasing 1/T, suggesting lower effective activation energies or greater system stability at higher antioxidant levels.

Table 4 presents the activation energies (E_a_), which range from approximately 14 to 33 kJ/mol depending on concentration. For the lowest concentration (111 μg/mL), E_a_ was 24.6 kJ/mol, whereas for 556 μg/mL it reached 32.8 kJ/mol. For 833 μg/mL, an intermediate value of 18.2 kJ/mol was obtained. For 278 μg/mL, after excluding the outlier at 140 °C, E_a_ calculated from the two lower temperatures was approximately 14.4 kJ/mol. These values indicate that α-tocopherol degradation is only moderately temperature-dependent, confirming its high thermal stability. Combined with zero-order kinetics, this means that α-tocopherol retains its protective activity across a wide range of conditions, consistent with the experimentally observed persistence of the absorbance signal even after prolonged heating.

Compared with β-carotene—characterized by lower activation energies and first-order kinetics—α-tocopherol behaves differently both in mechanism and temperature sensitivity. β-Carotene undergoes rapid, exponential decay, whereas α-tocopherol degrades more slowly and linearly over time. In practical terms, α-tocopherol functions as a more thermally robust antioxidant, capable of providing long-lasting protection to the lipid matrix under conditions where β-carotene has already undergone substantial decomposition. Understanding this distinction is crucial for designing lipid systems and oxidation-protection strategies.

**Figure 8:**
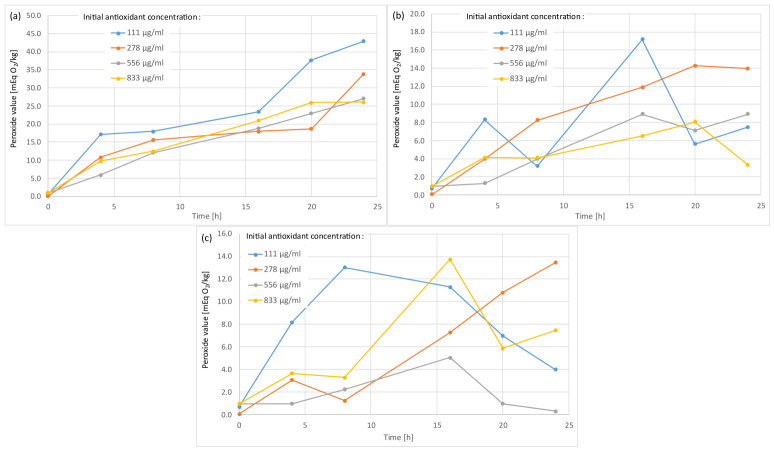
Changes in the peroxide value of FAME containing α-tocopherol over time at (**a**) 100 °C, (**b**) 120 °C, and (**c**) 140 °C.

**Figure 9:**
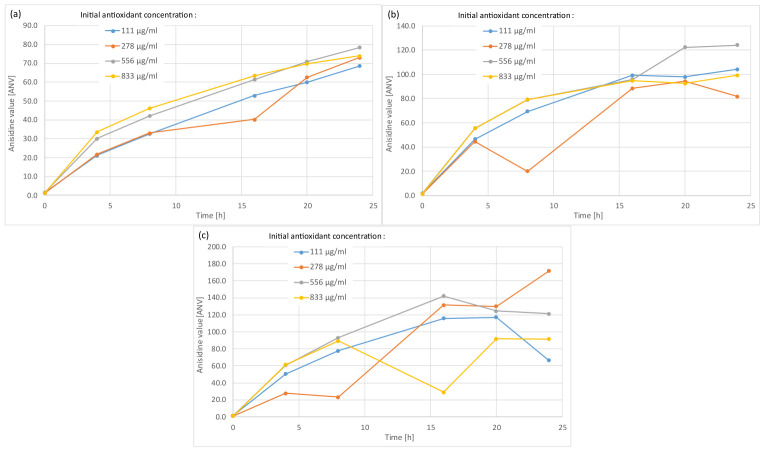
Changes in the anisidine value of FAME containing α-tocopherol over time at (**a**) 100 °C, (**b**) 120 °C, and (**c**) 140 °C.

The oxidative stability of FAME in the presence of α-tocopherol shows a clear dependence on both temperature and antioxidant dose. The dataset covering four α-tocopherol concentrations and three aging temperatures enables a detailed assessment of its effectiveness in inhibiting both early and advanced stages of oxidation. Changes in peroxide value (PV) and anisidine value (AnV), reflecting the formation of hydroperoxides and secondary oxidation products, respectively, are presented in Figure 8 and Figure 9.

At 100 °C, α-tocopherol exhibits a dose-dependent protective effect. After 24 h, PV reaches 42.92 meq O_2_/kg for 111 μg/mL, 33.90 for 278 μg/mL, 27.08 for 556 μg/mL, and 26.11 for 833 μg/mL, indicating that higher concentrations provide more effective inhibition of hydroperoxide formation. At 120 °C, PV increases more rapidly, and the protective effect becomes strongly dependent on concentration. The best stability is observed for 556 μg/mL, where PV remains low throughout the entire aging period. At 833 μg/mL, PVs are higher than for 556 μg/mL, suggesting that excessively high α-tocopherol concentrations may lead to pro-oxidant effects. At 140 °C, the differences between concentrations become even more pronounced. Again, 556 μg/mL provides the strongest protection, while 833 μg/mL shows irregular behavior indicative of instability or pro-oxidant activity.

Changes in AnV further support these observations. At 100 °C, AnV increases moderately for all concentrations, with relatively small differences between them. At 120 °C, the best performance is again observed for 556 μg/mL, while 833 μg/mL exhibits a sharp increase in AnV, confirming pro-oxidant behavior. At 140 °C, AnV remains relatively low for all concentrations, although 278–556 μg/mL provide the most effective suppression of aldehyde and ketone formation.

Overall, α-tocopherol is an effective antioxidant stabilizing FAME, with its efficiency depending on both dose and temperature. The best performance is observed for 556 μg/mL, which provides an optimal balance between thermal stability and radical-scavenging capacity. At elevated temperatures, α-tocopherol exhibits significantly greater stability than β-carotene, making it a more suitable additive for biofuels exposed to intense oxidative conditions. Excessively high doses (e.g., 833 μg/mL) may lead to pro-oxidant effects, indicating that optimal concentration ranges must be carefully defined.

In this study, activation energies for the degradation of β-carotene and α-tocopherol in the fatty acid methyl ester (FAME) matrix were determined. The obtained E_a_ values ranged from 6–23 kJ/mol for β-carotene and 14–33 kJ/mol or α-tocopherol. Such low activation energies are characteristic of systems with limited oxygen availability, high viscosity, and the absence of an aqueous phase, all of which significantly slow autoxidation processes. Consequently, both β-carotene and α-tocopherol exhibit a weaker temperature dependence of degradation rate in FAME than in typical food matrices.

According to the literature, β-carotene has been studied primarily in dried and encapsulated systems, where high susceptibility to oxidation results in much higher activation energies. The review by Lavelli and Sereikaitė [35] indicates that in powders and encapsulated systems, E_a_ for β-carotene typically ranges from 40–80 kJ/mol, due to intense oxidative degradation in matrices with large surface area exposed to air. Similar conclusions are reported in a comprehensive review on β-carotene degradation in fruit- and vegetable-based products, highlighting the strong influence of temperature, oxygen, and matrix structure on reaction rate and the high E_a_ values observed in aqueous and emulsion systems [36]. In oil-in-water emulsions, where β-carotene is particularly vulnerable to oxidation, high activation energies and rapid degradation rates are also observed, as confirmed by studies on carotenoid stability in the presence of antioxidants [37].

For α-tocopherol, activation energies in lipid matrices—such as vegetable oils or model systems—typically fall within 15–40 kJ/mol, consistent with the values obtained in this work. The stability of α-tocopherol arises from its phenolic antioxidant mechanism and its ability to scavenge lipid radicals, which slows its own degradation [21,38]. Reviews on the thermal degradation of antioxidant compounds emphasize that α-tocopherol belongs to a group of antioxidants with relatively high thermal resistance, and its E_a_ is lower than that of highly oxidation-prone compounds such as carotenoids [16,37,39].

The lower activation energies of β-carotene in FAME compared with literature values result primarily from the different matrix, where limited oxygen availability and the absence of an aqueous-phase slow autoxidation process are observed. α-tocopherol, on the other hand, exhibits E_a_ values consistent with literature data, confirming its high thermal stability in lipid systems and in FAME. These results are therefore fully consistent with current scientific knowledge and highlight the importance of matrix effects in interpreting the degradation kinetics of bioactive compounds.

In summary, β-carotene and α-tocopherol exhibit fundamentally different degradation behaviours in FAME. β-Carotene follows apparent first-order kinetics and undergoes rapid thermal decomposition above ~120 °C, which limits its antioxidant effectiveness to lower temperatures. In contrast, α-tocopherol degrades according to zero-order kinetics, retains its characteristic IR signal even after prolonged heating, and provides more sustained protection due to its higher thermal stability and different reaction pathways. These mechanistic differences explain the distinct temperature-dependent antioxidant performance observed for both compounds.

## 3. Materials and Methods

FAME were synthesized from refined rapeseed oil via classical transesterification with methanol in the presence of a NaOH catalyst, following procedures described by Grabowski et al. [40,41,42]. The reaction was carried out at 60 °C for 60 min using a 6:1 methanol-to-oil molar ratio, conditions considered optimal for high methyl ester yield [7]. After completion, the reaction mixture was allowed to separate by gravity, the glycerol phase was removed, and the FAME layer was washed with deionized water and dried under a nitrogen stream. The resulting product exhibited physicochemical properties consistent with typical methyl ester quality parameters (Table 5) and compliant with EN 14214 specifications [3]. The FAME sample was not required to meet all EN 14214 specification parameters, as it served as a model matrix for oxidation studies rather than as a commercial fuel. For mechanistic investigations of antioxidant activity, it is standard practice to use FAME that is chemically well-defined but not necessarily specification-compliant, provided that the matrix allows reproducible oxidation behavior. Only physicochemical parameters relevant to oxidation kinetics were determined, including density, viscosity, ester content, and initial PV and AnV.

β-carotene or α-tocopherol was dissolved directly in FAME to obtain solutions at 200 mg/L, following approaches commonly applied in oxidative stability studies of biofuels [11,19]. Samples for aging were prepared by dissolving β-carotene or α-tocopherol solution in FAME to obtain final concentrations of 13, 36, 58 and 71 µg/mL (β-carotene) and 111, 278, 556 and 833 µg/mL (α-tocopherol). Stock solutions were prepared by weighing the antioxidant and dissolving it in a small aliquot of FAME, then diluting to the final volume. Each solution was vortexed for 2 min and sonicated for 5 min (Ultrasonic bath, JPS-08A, Vevor, Shanghai, China) to ensure homogeneity. Samples (15 mL) were transferred into 20 mL borosilicate vials and filled to 75% of vial volume, leaving ~5 mL headspace to standardize oxygen availability, in accordance with recommendations for thermo-oxidative aging studies [16,24]. Vials were loosely capped (to allow gas exchange) and placed in a heating block (HB2DG, Ohaus, Zurich, Switzerland) with temperature stability ±0.5 °C.

The concentrations of β-carotene (13–71 µg/mL) and α-tocopherol (111–833 µg/mL) were selected based on their solubility in FAME and on ranges commonly reported in studies of natural antioxidants in lipid matrices. For β-carotene, the upper limit was determined by its solubility and the requirement to maintain linear absorbance in UV–Vis measurements. α-Tocopherol, being substantially more soluble, was tested at proportionally higher concentrations to achieve comparable molar antioxidant-to-substrate ratios. This design enabled the evaluation of both low and high antioxidant doses compared to naturally occurring FAME from unrefined rapeseed oil. Ensured that concentration-dependent effects could be reliably assessed under thermo-oxidative conditions.

Thermo-oxidation was conducted at 100, 120, and 140 °C, temperatures representative of accelerated aging conditions for FAME [17,24]. Samples were heated for 4, 8, 16, 20, and 24 h. After heating, vials were cooled to room temperature in a desiccator prior to analysis. The thermo-oxidation experiments were conducted at 100–140 °C, which corresponds to a standard accelerated-aging regime commonly used for biodiesel and edible oils. This temperature range enables the observation of oxidation pathways and antioxidant behavior within practical laboratory timescales, as changes occurring at ambient temperature would require weeks or months to become measurable. Although such temperatures do not reflect typical storage conditions, short-term thermal stress may occur in fuel systems (e.g., injector recirculation, heated return lines or filtration units). The purpose of applying elevated temperatures in this study was therefore to obtain mechanistic insight into the thermo-oxidative degradation of FAME in the presence of β-carotene and α-tocopherol. This accelerated thermo-oxidation regime was applied to obtain mechanistic information within practical experimental timescales, complementing long-term storage studies performed at ambient temperature. Oxidative stability was assessed using peroxide value (PV) and anisidine value (AnV), standard indicators of primary and secondary oxidation products in lipid and biofuel systems [18,25,40]. PV was determined iodometrically according to EN-ISO 3960 [43], while AnV was measured according to EN-ISO 6885 [44].

Degradation of β-carotene and α-tocopherol was monitored by UV–Vis and FT-IR spectroscopy following established methodologies for natural antioxidants in FAME matrices [30]. UV–Vis spectra were recorded using a UV-1800 spectrophotometer [Shimadzu, Kyoto, Japan] with 1 cm pathlength quartz cuvettes; FT-IR spectra were recorded using a FT-IR Invenio spectrometer [Brucker, Ettlingen, Germany] with a fixed-path liquid cell (pathlength 1 mm). UV–Vis spectra were recorded over 200–700 nm, with changes in the characteristic β-carotene absorption band at 450 nm evaluated [45]. FT-IR spectra were recorded using a fixed-path cuvette in the 4000–400 cm^−1^ range, with particular attention to the band at 1068 cm^−1^ corresponding to phenolic –OH vibrations in α-tocopherols (C-O bond in phenols) [40,46]. These spectral features (450 nm for β-carotene and 1068 cm^−1^ for α-tocopherol) were used as analytical maxima for quantitative evaluation. In kinetic analyses, absorbance values were used directly, as absorbance is proportional to concentration according to the Beer–Lambert law; conversion to concentration was avoided to prevent additional error. The choice of UV–Vis for β-carotene and FT-IR for α-tocopherol reflects the distinct spectroscopic characteristics of these antioxidants, ensuring optimal selectivity and sensitivity for each compound. These techniques were also selected because they are widely accessible and easily reproducible in routine and industrial laboratory settings, which enhances the practical applicability of the analytical approach.

All measurements were performed in triplicate, and results are reported as mean values. Statistical analysis was conducted using ANOVA at a significance level of *p* < 0.05, consistent with common practice in oxidative stability studies of biofuels and vegetable oils [41,42]. This methodology enabled a comprehensive assessment of both methyl ester oxidation kinetics and the thermal stability of β-carotene and α-tocopherol, as well as the relationship between antioxidant degradation and protective efficacy, in line with current research trends in biofuel stabilization [14,19,27].

Kinetic fits were performed by ordinary least squares regression using [Microsoft^®^ Excel^®^ for Microsoft 365 MSO, version 2605]. For β-carotene, first-order kinetics were tested by linear regression of ln(*A*) vs. *t*. In addition to full-time fits, initial rates were estimated by linear regression of *A* vs. *t* over the first 2 h to capture early-time behavior. Model selection was based on coefficient of determination (R^2^), residual analysis and *p*-values (α = 0.05). Normalized plots *A*/*A*_0_ were inspected to compare relative (fractional) decay across concentrations independently of absolute absorbance.

All reagents were used without further purification immediately after purchase from local chemical suppliers.

## 4. Conclusions

The oxidative stabilization of fatty acid methyl esters depends fundamentally on the chemical nature, thermal resistance, and reaction mechanisms of the antioxidant used. The comparison of β-carotene and α-tocopherol reveals two distinctly different behavioral models, leading to clear conclusions regarding their suitability for protecting FAME.

β-Carotene exhibits antioxidant activity only under moderate thermal conditions, where singlet-oxygen quenching and radical scavenging can delay the initiation of oxidation. At 100 °C, a short-lived protective effect is observed, particularly at higher concentrations. However, at 120–140 °C, β-carotene undergoes rapid degradation, resulting in the loss of activity and the formation of secondary products that may exert pro-oxidant effects. Its applicability is therefore limited to low-temperature environments and short exposure times.

α-Tocopherol demonstrates a fundamentally different protective behavior. Its high thermal stability, arising from the formation of stable tocopheryl radicals, enables effective interruption of chain reactions even at elevated temperatures. The results show that α-tocopherol at 556 μg/mL provides optimal protection across the entire temperature range studied, maintaining low peroxide and anisidine values after 24 h of aging. Lower concentrations (111–278 μg/mL) offer limited protection, whereas excessive amounts (833 μg/mL) may lead to pro-oxidant effects, particularly at 120 °C. Unlike β-carotene, α-tocopherol does not lose activity at high temperature and does not generate degradation products that accelerate oxidation.

Overall, α-tocopherol is a far more effective and thermally stable antioxidant for FAME than β-carotene. Its predictable, long-lasting behavior across a wide temperature range makes it a practical choice for biodiesel stabilization. β-Carotene may serve as a supplementary antioxidant, but only under mild conditions where thermal degradation is minimal. For industrial applications—particularly for FAME produced from refined rapeseed oil—α-tocopherol remains the superior antioxidant additive.

## Figures and Tables

**Table 1:** Rate constants and corresponding half-life values for the degradation of β-carotene in FAME.

Temperature, °C	Rate Constant k, h^−1^	Half-Life t_1_/_2_, h
100	0.11	6.5
120	0.14	5.1
140	0.13	5.4

**Table 2:** Activation energy of β-carotene degradation in FAME.

Concentration, μg/mL	13	35	58	71
E_a_, kJ/mol	6.0	14.9	12.6	22.8

**Table 3:** Rate constants and corresponding half-life values for the degradation of α-tocopherol in FAME.

Temperature, °C	Concentration, μg/mL	Rate Constant k, h^−1^	Half-Life t_1_/_2_, h
100	111	5.86 × 10^−4^	243.33
	278	7.41 × 10^−4^	186.71
	556	5.06 × 10^−4^	271.03
	833	4.11 × 10^−4^	326.53
120	111	5.90 × 10^−4^	235.94
	278	9.36 × 10^−4^	146.41
	556	10.18 × 10^−4^	134.39
	833	5.87 × 10^−4^	228.81
140	111	12.62 × 10^−4^	108.76
	278	0.37 × 10^−4^ *	3419.09 *
	556	14.08 × 10^−4^	96.03
	833	7.25 × 10^−4^	184.92

* Excluded from kinetic fitting due to non-monotonic A(t) behavior.

**Table 4:** Activation energy of α-tocopherol degradation in FAME.

Concentration, μg/mL	111	278	556	833
E_a_, kJ/mol	24.6	14.4	32.8	18.2

**Table 5:** Physico-chemical properties of the FAME prepared from unrefined rapeseed oil.

Parameters	FAME	Norm Values [3]
Density in 15 °C, kg/m^3^	893 ± 3	860–900
Kinematic viscosity in 40 °C, mm^2^/s	4.55 ± 0.24	3.50–5.00
Total concentration of methyl esters, % *v*/*v*	95.8 ± 0.8	min. 96.5
Oxidation stability, h	1.0 ± 0.2	min. 8.0
Peroxide number, meq O_2_/kg	0.53 ± 0.11	Non-normative parameter
Anisidine number, AnV	0.63 ± 0.13	Non-normative parameter
Acid number, mg KOH/g	1.50 ± 0.24	max. 0.5
Color	light yellow	Non-normative parameter
β-carotene concentration, μg/mL	<LLD ^1^	Non-normative parameter
α-tocoferol concentration, μg/mL	<LLD ^2^	Non-normative parameter

^1^ The LLD for the analysis by the UV–Vis method was 1.51 μg/mL. ^2^ The LLD for the analysis by the FTIR method was 0.23 μg/mL.

## Data Availability

The original contributions presented in this study are included in the article. Further inquiries can be directed to the corresponding author.

## Abbreviations

The following abbreviations are used in this manuscript:

FAME	Fatty Acid Methyl Esters
UV-Vis spectroscopy	Ultraviolet-Visible spectroscopy
FT-IR spectroscopy	Fourier Transfer-InfraRed spectroscopy
RED	Renewable Energy Directive
EN	European Norm
PV	Peroxide Value
AnV	Anisidine Value
AV	Acid Value
SPLET	Sequential Proton Loss Electron Transfer
LLD	Low Limit of Detection
ANOVA	Analysis of Variance
E_a_	Activation energies
IR-band	Infrared band

## References

[1] European Parliament and Council (2018). Directive (EU) 2018/2001 on the Promotion of the Use of Energy from Renewable Sources (RED II). Off. J. Eur. Union.

[2] European Parliament and Council (2023). Directive (EU) 2023/2413 amending Directive (EU) 2018/2001, Regulation (EU) 2018/1999 and Directive 98/70/EC as regards the promotion of energy from renewable sources, and repealing Council Directive (EU) 2015/652 (RED III). Off. J. Eur. Union.

[3] (2019). Liquid Petroleum Products—Fatty Acid Methyl Esters (FAME) for Use in Diesel Engines and Heating Applications—Requirements and Test Methods.

[4] European Parliament and Council (2009). Directive 2009/30/EC amending Directive 98/70/EC relating to the quality of petrol and diesel fuels (Fuel Quality Directive). Off. J. Eur. Union.

[5] Knothe G. (2005). Dependence of biodiesel fuel properties on the structure of fatty acid alkyl esters. Fuel Process. Technol..

[6] Ramos M.J., Fernández C.M., Casas A., Rodríguez L., Pérez Á. (2009). Influence of fatty acid composition of raw materials on biodiesel properties. Bioresour. Technol..

[7] Atabani A., Silitonga A., Ong H., Mahlia T., Masjuki H., Badruddin I.A., Fayaz H. (2013). Non-edible vegetable oils: A critical evaluation of oil extraction, fatty acid compositions, biodiesel production, characteristics, engine performance and emissions production. Renew. Sustain. Energy Rev..

[8] Mittelbach M., Schober S. (2003). The influence of antioxidants on the oxidation stability of biodiesel. J. Am. Oil Chem. Soc..

[9] Jain S., Sharma M.P. (2010). Oxidation Stability of Biodiesel and Its Blends: A Review. Renew. Sustain. Energy Rev..

[10] Knothe G. (2007). Some aspects of biodiesel oxidative stability. Fuel Process. Technol..

[11] Dunn R.O. (2007). Effect of Temperature on the Oil Stability Index (OSI) of Biodiesel. Energy Fuels.

[12] Bouaid A., Martinez M., Aracil J. (2007). Long storage stability of biodiesel from vegetable and used frying oils. Fuel.

[13] Focke W.W., van der Westhuizen I., Grobler A.L., Nshoane K.T., Reddy J.K., Luyt A.S. (2012). The effect of synthetic antioxidants on the oxidative stability of biodiesel. Fuel.

[14] Varatharajan K., Pushparani D. (2018). Screening of antioxidant additives for biodiesel fuels. Renew. Sustain. Energy Rev..

[15] Jain S., Sharma M. (2011). Thermal stability of biodiesel and its blends: A review. Renew. Sustain. Energy Rev..

[16] Kamal-Eldin A., Appelqvist L.-Å. (1996). The chemistry and antioxidant properties of tocopherols and tocotrienols. Lipids.

[17] Brigelius-Flohé R., Traber M.G. (1999). Vitamin E: Function and metabolism. FASEB J..

[18] Shahidi F., Zhong Y. (2010). Lipid oxidation and improving the oxidative stability. Chem. Soc. Rev..

[19] Britton G. (1995). Structure and properties of carotenoids in relation to function. FASEB J..

[20] Stahl W., Sies H. (2003). Antioxidant Activity of Carotenoids. Mol. Asp. Med..

[21] Choe E., Min D.B. (2006). Mechanisms and Factors for Edible Oil Oxidation. Compr. Rev. Food Sci. Food Saf..

[22] Mittelbach M., Gangl S. (2001). Long storage stability of biodiesel made from rapeseed and used frying oil. J. Am. Oil Chem. Soc..

[23] de Sousa L.S., de Moura C.V.R., de Oliveira J.E., de Moura E.M. (2014). Use of natural antioxidants in soybean biodiesel. Fuel.

[24] Tang H., De Guzman R.C., Ng K.Y.S., Salley S.O. (2009). Effect of Antioxidants on the Storage Stability of Soybean-Oil-Based Biodiesel. Energy Fuels.

[25] Rodriguez-Amaya D.B., Kimura M. (2004). HarvestPlus Handbook for Carotenoid Analysis.

[26] Boon C.S., McClements D.J., Weiss J., Decker E.A. (2010). Factors Influencing the Chemical Stability of Carotenoids in Foods. Crit. Rev. Food Sci. Nutr..

[27] Galano A., Vargas R., Martínez A. (2009). Carotenoids can act as antioxidants by oxidizing the superoxideradical anion. Phys. Chem. Chem. Phys..

[28] Wang S., Meckling K.A., Marcone M.F., Kakuda Y., Tsao R. (2011). Synergistic, Additive, and Antagonistic Effects of Food Mixtures on Total Antioxidant Capacities. J. Agric. Food Chem..

[29] Noon J., Mills T.B., Norton I.T. (2020). The use of natural antioxidants to combat lipid oxidation in O/W emulsions. J. Food Eng..

[30] Choe E., Min D.B. (2009). Mechanisms of Antioxidants in the Oxidation of Foods. Compr. Rev. Food Sci. Food Saf..

[31] Encinar J., Sánchez N., Martínez G., García L. (2011). Study of biodiesel production from animal fats with high free fatty acid content. Bioresour. Technol..

[32] Kamal-Eldin A. (2003). Lipid Oxidation Pathways.

[33] Schaich K.M., Akoh C.C. (2017). Rethinking lipid oxidation. Food Lipids Chemistry, Nutrition, and Biotechnology.

[34] Dunn R.O. (2005). Effect of antioxidants on the oxidative stability of methyl soyate (biodiesel). Fuel Process. Technol..

[35] Lavelli V., Sereikaitė J. (2022). Kinetic Study of Encapsulated β-Carotene Degradation in Aqueous Environments: A Review. Foods.

[36] Pénicaud C., Achir N., Dhuique-Mayer C., Dornier M., Bohuon P. (2011). Degradation of β-carotene during fruit and vegetable processing or storage: Reaction mechanisms and kinetic aspects: A review. Fruits.

[37] Desobry S.A., Netto F.M., Labuza T.P. (1997). Comparison of Spray-drying, Drum-dryingand Freeze-drying for b-CaroteneEncapsulation and Preservation. J. Food Sci..

[38] Romola C.J., Meganaharshini M., Rigby S., Moorthy I.G., Kumar R.S., Karthikumar S. (2021). A comprehensive review of the selection of natural and synthetic antioxidants to enhance the oxidative stability of biodiesel. Renew. Sustain. Energy Rev..

[39] Man Y.B.C., Ammawath W., Mirghani M.E.S. (2005). Determining α-tocopherol in refined bleached and deodorized palm olein by Fourier transform infrared spectroscopy. Food Chem..

[40] Grabowski P., Szwarczyńska A. (2024). Non-Normative Oxidation Stability Indication of FAME Produced from Rapeseed and Used Cooking Oil. Energies.

[41] Grabowski P., Nowakowska A. (2024). Anisidine value as oxidation stability indicator in FAME. Biofuels.

[42] Wilińska I., Grabowski P., Słoński M., Koc M. (2026). The Influence of Raw Materials for Fatty Acid Methyl Ester Production on the Aging Rate of Diesel Fuel Blends with Biocomponents. Energies.

[43] (2017). Animal and Vegetable Fats and Oils—Determination of Peroxide Value—Iodometric (Visual) Endpoint Determination.

[44] (2016). Animal and Vegetable Fats and Oils—Determination of Anisidine Value.

[45] Safdarian M., Hashemi P., Ghiasvand A. (2021). A fast and simple method for determination of β-carotene in commercial fruit juice by cloud point extraction-cold column trapping combined with UV–Vis spectrophotometry. Food Chem..

[46] Silva S.D., Rosa N.F., Ferreira A.E., Boas L.V., Bronze M.R. (2008). Rapid Determination of α-Tocopherol in Vegetable Oils by Fourier Transform Infrared Spectroscopy. Food Anal. Methods.

